# Supplementary material for: A proximity biotinylation-based approach to identify protein-E3 ligase interactions induced by PROTACs and molecular glues
Source: Nat Commun. 2022 Jan 10;13:183. doi: 10.1038/s41467-021-27818-z (PMC8748630; doi:10.1038/s41467-021-27818-z)
Supplement: Supplementary file 1 — Supplementary Information [file 41467_2021_27818_MOESM1_ESM.pdf]

Supplementary Information:

A proximity biotinylation-based approach to identify protein-E3 ligase interactions induced by PROTACs and molecular glues

Satoshi Yamanaka[1], Yuto Horiuchi[1], Saya Matsuoka[1], Kohki Kido[1], Kohei Nishino[2], Mayaka Maeno[3], Norio Shibata[3], Hidetaka Kosako[2], Tatsuya Sawasaki[1*]

[1]Division of Cell-Free Sciences, Proteo-Science Center, Ehime University, Matsuyama, 790-8577 Japan, [2]Division of Cell Signaling, Fujii Memorial Institute of Medical Sciences, Tokushima University, Tokushima 770-8503, Japan, [3]Department of Nanopharmaceutical Sciences, Nagoya Institute of Technology, Nagoya, 466-8555, Japan.

*Corresponding Author:
Tatsuya Sawasaki
Proteo-Science Center,
Ehime University, Matsuyama 790-8577, Japan
Tel: 81-89-927-8530
Fax: 81-89-927-9941
E-mail address: sawasaki@ehime-u.ac.jp.

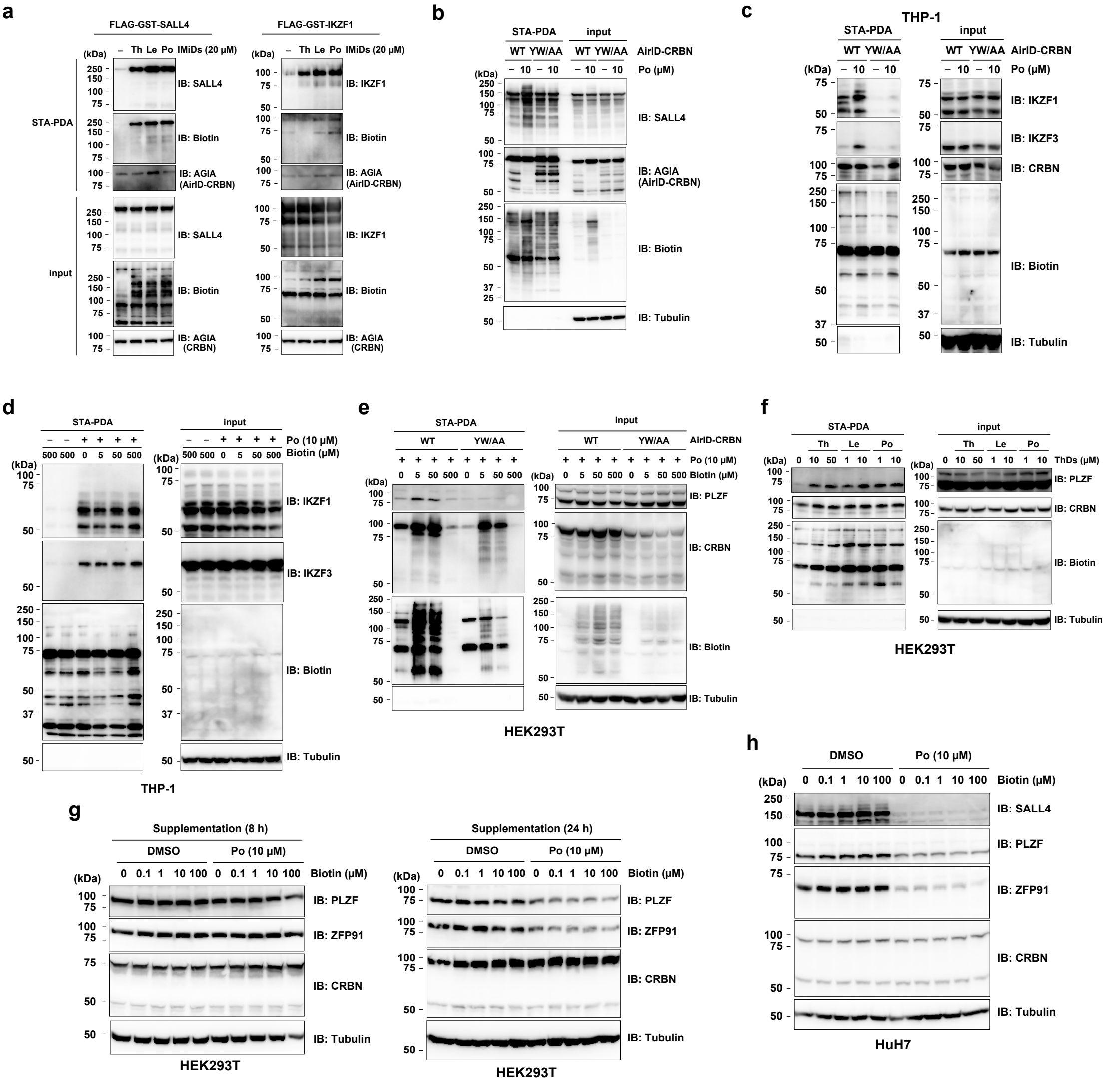

Supplementary Fig. 1. Optimization of thalidomide and its derivatives (IMiDs)-dependent neo-substrate biotinylation. a, *In vitro* IMiD-dependent biotinylation assay of neo-substrates by AirID-CRBN. Biotinylation of FLAG-GST-SALL4 or -IKZF1 by AirID-CRBN was performed in the presence of DMSO, thalidomide (Th), lenalidomide (Le) or pomalidomide (Po) at 26 °C for 3 h. **b,** IMiD-dependent biotinylation assay of exogenous neo-substrates by AirID-CRBN in cells. HEK293T cells were transfected with AGIA-AirID-CRBN-WT or -YW/AA and Myc-SALL4 and treated with DMSO or 10 μM pomalidomide (Po) and 10 μM biotin and 5 μM MG132 for 6 h. **c,** IMiD-dependent biotinylation assay of endogenous neo-substrate by AirID-CRBN in THP-1 cells. THP-1 cells stably expressing AGIA-AirID-CRBN-WT or -YW/AA were treated with DMSO or 10 μM pomalidomide (Po) in the presence of 10 μM biotin and 5 μM MG132 for 8 h. **d,** Biotin dose-dependent biotinylation assay of endogenous neo-substrates by AirID-CRBN in THP-1 cells. THP-1 cells stably expressing AGIA-AirID-CRBN-WT were treated with DMSO or 10 μM pomalidomide (Po) in the presence of 0, 5, 50 or 500 μM biotin and 5 μM MG132 for 8 h. **e,** Biotin dose-dependent biotinylation assay of endogenous neo-substrate by AirID-CRBN in HEK293T cells. HEK293T cells stably expressing AGIA-AirID-CRBN-WT or -YW/AA were treated with DMSO or 10 μM pomalidomide (Po) in the presence of 0, 5, 50 or 500 μM biotin and 5 μM MG132 for 8 h. **f,** IMiD dose-dependent biotinylation assay of endogenous neo-substrates by AirID-CRBN in HEK293T cells. HEK293T cells stably expressing AGIA-AirID-CRBN-WT were treated with DMSO, thalidomide (Th), lenalidomide (Le) or pomalidomide (Po) in the presence of 10 μM biotin and 5 μM MG132 for 8 h. **g,** Immunoblot analysis of neo-substrates protein levels in HEK293T cells treated with DMSO or

pomalidomide (Po) in the presence of biotin for 8 h or 24 h. **h,** Immunoblot analysis of neo-substrates protein levels in HEK293T cells treated with DMSO or pomalidomide (Po) in the presence of biotin for 8 h. **a–f,** Biotinylated proteins were pulled down using streptavidin beads and analysed by immunoblotting. All experiments were repeated twice independently with similar results. Source data are provided as a Source data file.

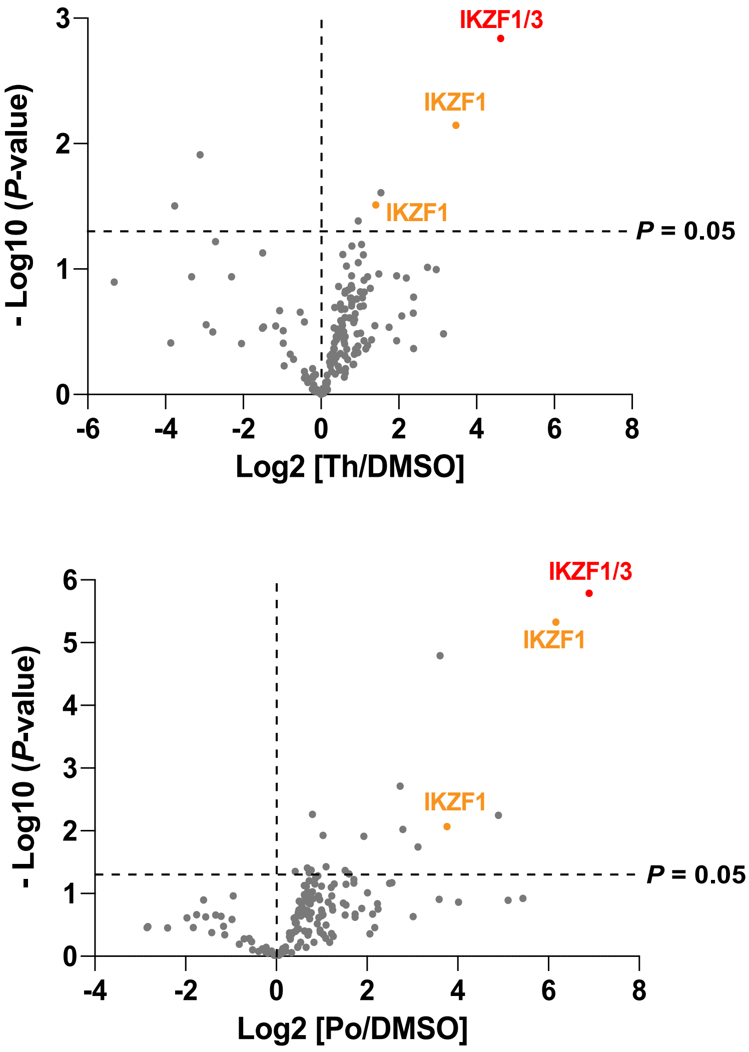

Supplementary Fig. 2. LC-MS/MS analysis of biotinylated peptides using AirID-CRBN in THP-1 cells. THP-1 cells stably expressing AGIA-AirID-CRBN-WT were treated with DMSO, 20 µM thalidomide (Th) or 10 µM pomalidomide (Po) in the presence of 10 µM biotin and 5 µM MG132 for 8 h (biological replicates; n = 3). Then, the biotinylated peptides were enriched with tamavidin 2-REV followed by LC-MS/MS analysis. Significant changes in the volcano plots were calculated by Student's two-sided *t*-test, and the false discovery rate (FDR)-adjusted *P*-values calculated using Benjamini-Hochberg method are shown in the Supplementary Data 1.

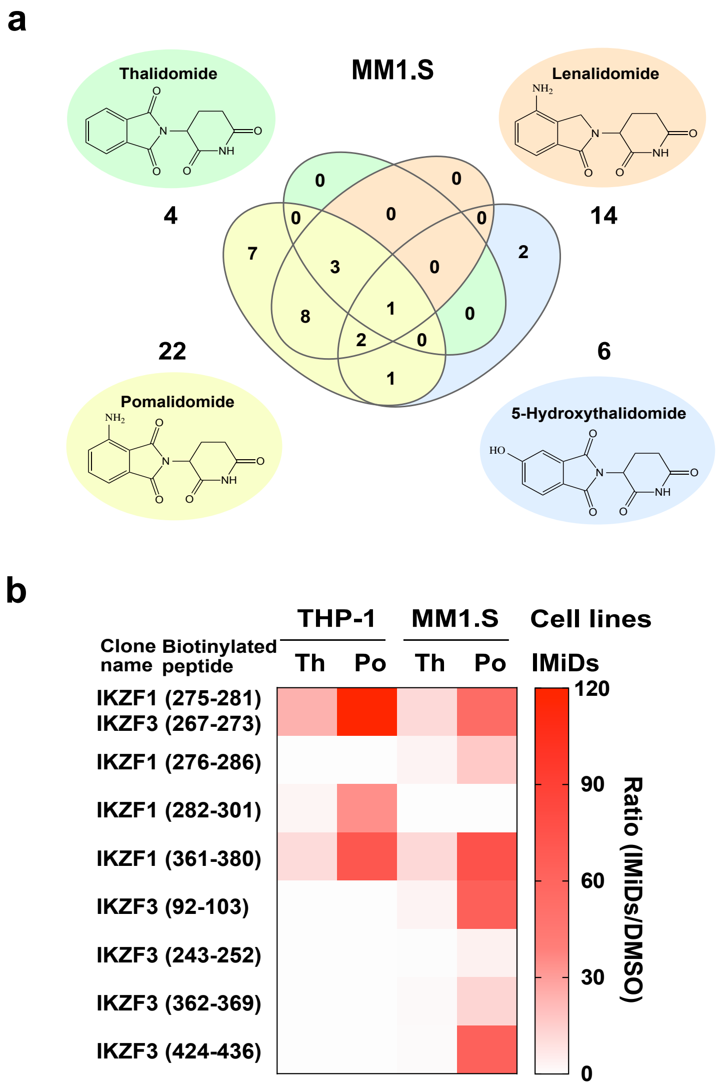

Supplementary Fig. 3. Comparison of thalidomide and its derivatives (IMiDs)-inducible biotinylation from LC-MS/MS analysis in THP-1 and MM1.S cells. a, Comparison of biotinylated peptides among IMiDs detected by LC-MS/MS analysis. Overlapping IMiD-dependent biotinylated peptides (IMiD/DMSO ratio > 5) in MM1.S cells were compared among IMiDs using a Venn diagram. **b,** Comparison of biotinylated peptides of IKZF1 or IKZF3 (IMiD/DMSO ratio > 5 and *P*-value < 0.05) between THP1 cells and MM1.S cells on a heat map. Significant changes in the heatmap were calculated by Student's two-sided *t*-test and the false discovery rate (FDR)-adjusted *P*-values calculated using Benjamini-Hochberg method are shown in the Supplementary Data 1, 5. Source data are provided as a Source data file.

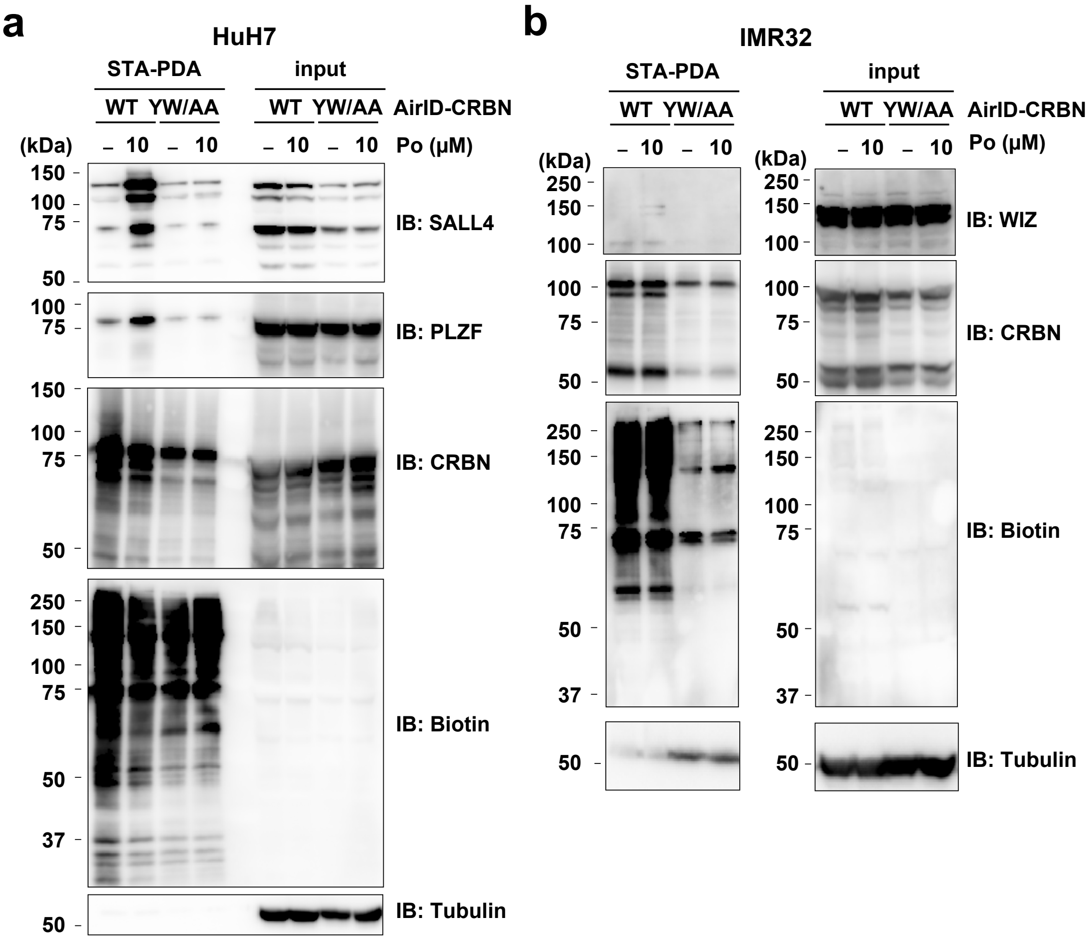

Supplementary Fig. 4. Streptavidin pull-down assays in HuH7 and IMR32 cells expressing AirID-CRBN wild type or AirID-CRBN mutant. a-b, Thalidomide and its derivatives (IMiDs)-dependent biotinylation assay of known neo-substrates via STA-PD assay. (**a**) HuH7 or (**b**) IMR32 cells stably expressing AGIA-AirID-CRBN-WT or -YW/AA were treated with DMSO or 10 μM pomalidomide (Po) in the presence of 10 μM biotin and 5 μM MG132 for 8 h. Then, the biotinylated proteins were pulled down using streptavidin beads and analysed by immunoblotting. The experiments were repeated three times independently with similar results. Source data are provided as a Source data file.

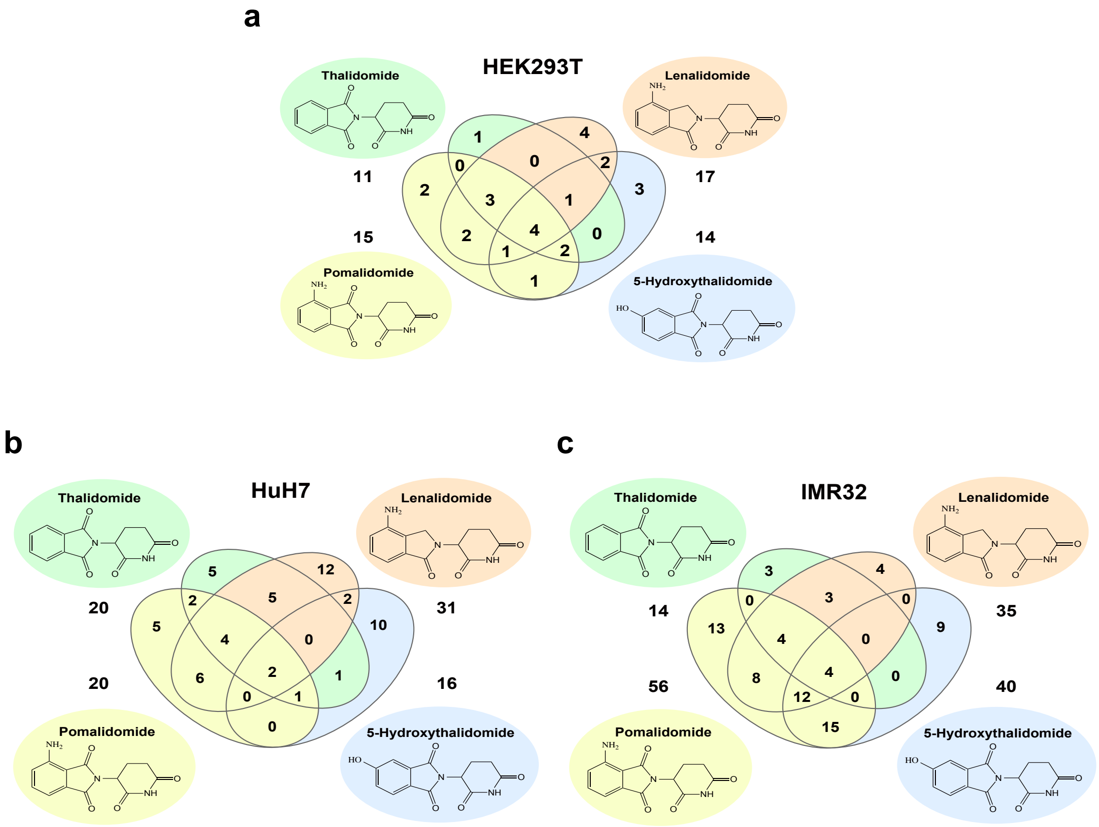

Supplementary Fig. 5. Comparison of LC-MS/MS analyses from HEK293T, HuH7, or IMR32 cells with thalidomide and its derivatives (IMiDs). a, Comparison of biotinylated peptides among IMiDs detected by LC-MS/MS analysis using HEK293T cells. Overlapping IMiD-dependent biotinylated peptides (IMiD/DMSO ratio > 5) were compared among IMiDs using a Venn diagram. **b-c,** Comparison of biotinylated peptides among IMiDs detected by LC-MS/MS analysis using (**b**) HuH7 or (**c**) IMR32 cells. Overlapping IMiD-dependent biotinylated peptides (IMiD/DMSO ratio > 5) were compared among IMiDs using a Venn diagram.

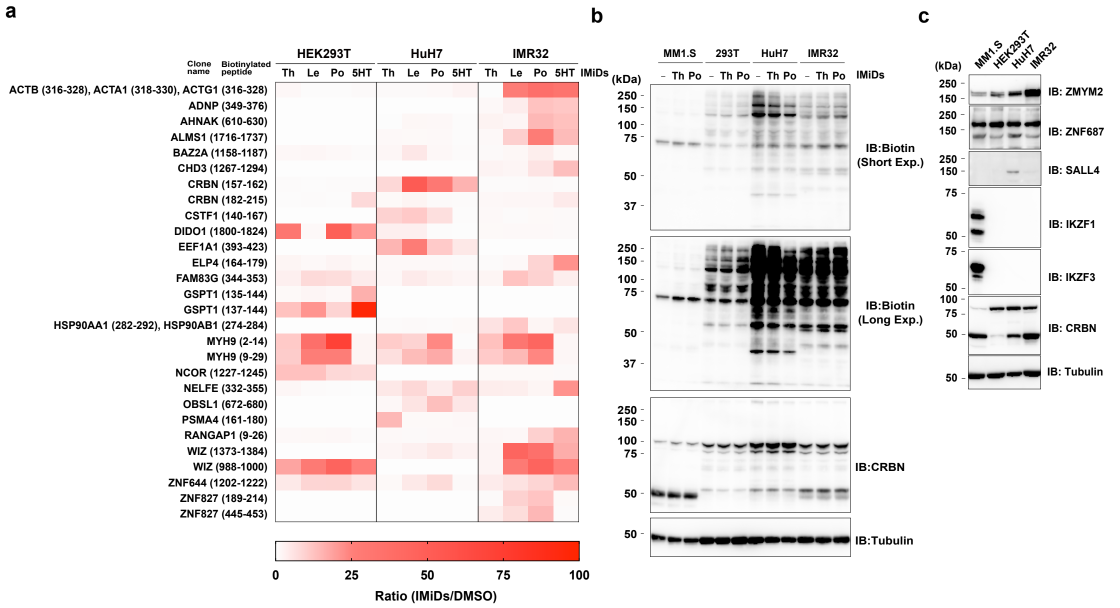

Supplementary Fig. 6. Comparison of thalidomide and its derivatives (IMiDs)-inducible biotinylated peptides by AirID-CRBN among cell lines. a, Comparison of biotinylated peptides (IMiD/DMSO ratio > 10 and *P*-value < 0.05) among HEK293T, HuH7, and IMR32 cells on a heat map. Significant changes in the heatmap were calculated by Student's two-sided *t*-test and the false discovery rate (FDR)-adjusted *P*-values calculated using Benjamini-Hochberg method are shown in the Supplementary Data 6–8. **b,** Biotinylated peptides among MM1.S, HEK293T, HuH7, and IMR32 cells treated with DMSO, 10 µM thalidomide (Th) or 10 µM pomalidomide (Po) in the presence of 10 µM biotin and 5 µM MG132 were compared by immunoblot analysis. The experiment was repeated three times independently with similar results. **c,** Protein-expression levels of neo-substrates among MM1.S, HEK293T, HuH7 and IMR32 cells were compared by immunoblotting. The experiment was repeated three times independently with similar results. Source data are provided as a Source data file.

a

	MM1.S	293T	HuH7	IMR32
CUL4A	○	○	○	○
CUL4B	×	○	○	○
DDB1	○	×	○	○
RBX1	×	×	×	×
UBC12	×	○	○	○
CSN	○	○	○	○
UBE2D3	×	×	×	×
UBE2G1	×	×	×	×

b

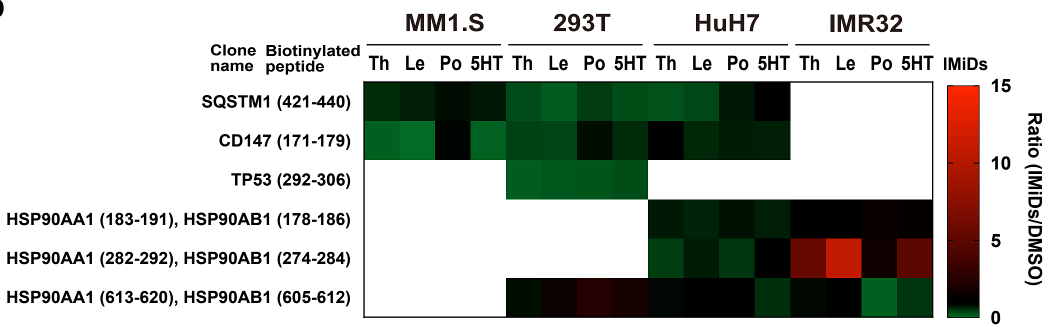

Supplementary Fig. 7. Table and heatmap of known proteins involved in CRL4^{CRBN} and proteins known to interact with CRBN. a, Table of known proteins involved in CRL4^{CRBN} in LC-MS/MS analyses in MM1.S, HEK293T, HuH7 and IMR32 cells. **b,** Comparison of biotinylated peptides from proteins known to interact with CRBN among HEK293T, HuH7 and IMR32 cells on a heat map. Source data are provided as a Source data file.

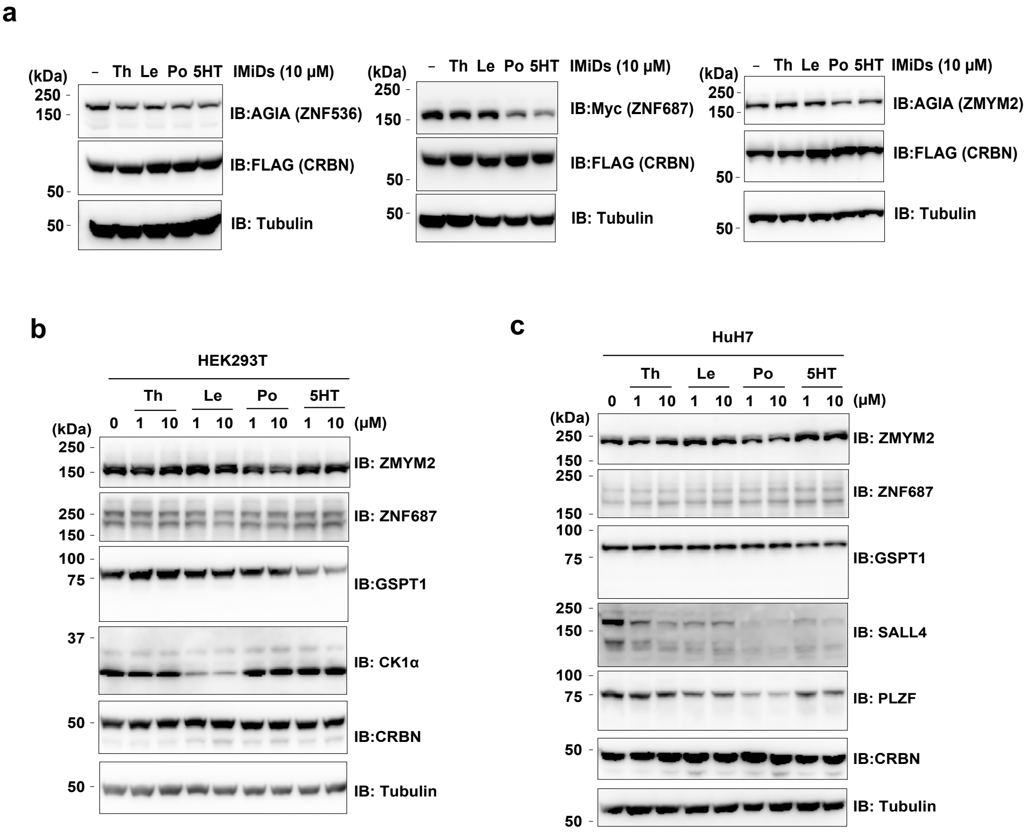

Supplementary Fig. 8. Protein degradations of neo-substrate candidates identified by LC-MS/MS analysis and biotinylated peptide analyses. a, Immunoblot analysis of thalidomide and its derivatives (IMiDs)-dependent protein degradation of ZNF536, ZNF687, and ZMYM2. HEK293T cells expressing AGIA-ZNF536, Myc-ZNF687 or ZMYM2-AGIA, and FLAG-CRBN were treated with DMSO, thalidomide (Th), lenalidomide (Le), pomalidomide (Po) or 5-hydroxythalidomide (5HT) for 16 h. The experiment was repeated three times independently with similar results. **b-c,** Immunoblot analysis of endogenous ZMYM2, ZNF687, GSPT1, CK1α, GSPT1, SALL4 and PLZF protein levels in (**b**) HEK293T cells or (**c**) HuH7 cells treated with DMSO, thalidomide (Th), lenalidomide (Le), pomalidomide (Po) or 5-hydroxythalidomide (5HT) for 24 h. The experiment was repeated three times independently with similar results. Source data are provided as a Source data file.

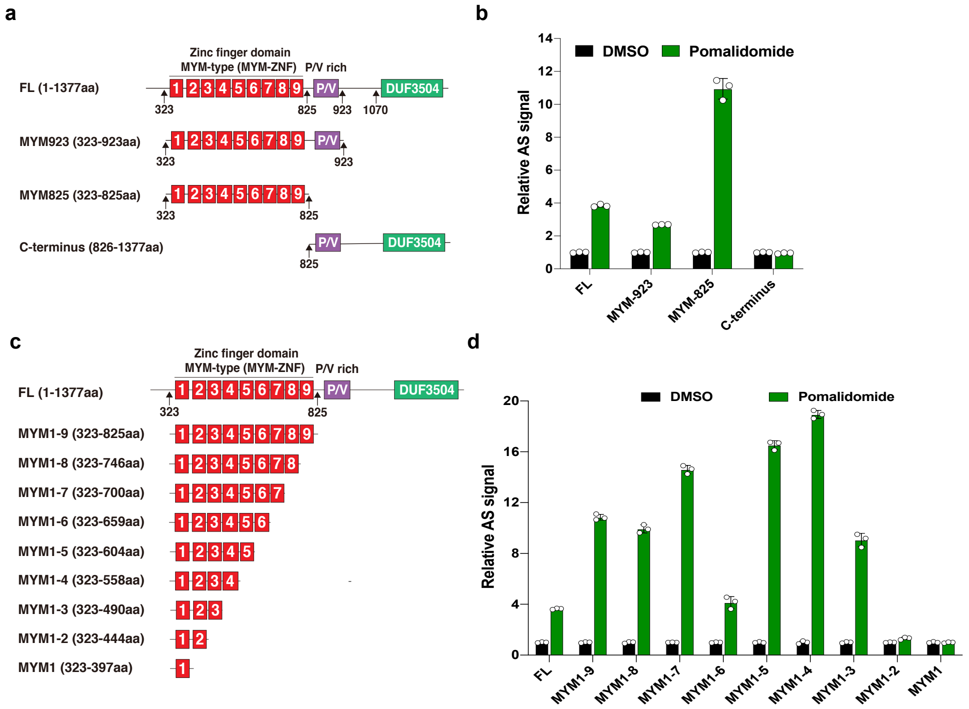

Supplementary Fig. 9. Third MYM-ZNF is required for pomalidomide (Po)-dependent interaction between ZMYM2 and CRBN. a, Schematic diagram of truncated ZMYM2 protein. FL; full-length. **b,** *In vitro* interaction assay between CRBN and truncated ZMYM2. The interaction between bls-CRBN and FLAG-GST-ZMYM2-FL, -MYM923, -MYM825 or -C-terminus in the presence of DMSO or 20 µM Po was analysed using the AlphaScreen-based biochemical assay. Error bars denote the mean ± standard deviation (independent experiments; n = 3). **c,** Schematic diagram of truncated MYM-ZNF of ZMYM2. **d,** *In vitro* interaction assay between CRBN and truncated MYM-ZNFs. The interaction between biotin-labeled bls-CRBN and FLAG-GST-ZMYM2-FL or -truncated MYM in the presence of DMSO or 20 µM pomalidomide (Po) was analysed using the AlphaScreen-based biochemical assay. Error bars denote the mean ± standard deviation (independent experiments; n = 3). **b, d,** All relative AlphaScreen (AS) signals are expressed as luminescence signals relative to that of DMSO. Source data are provided as a Source data file.

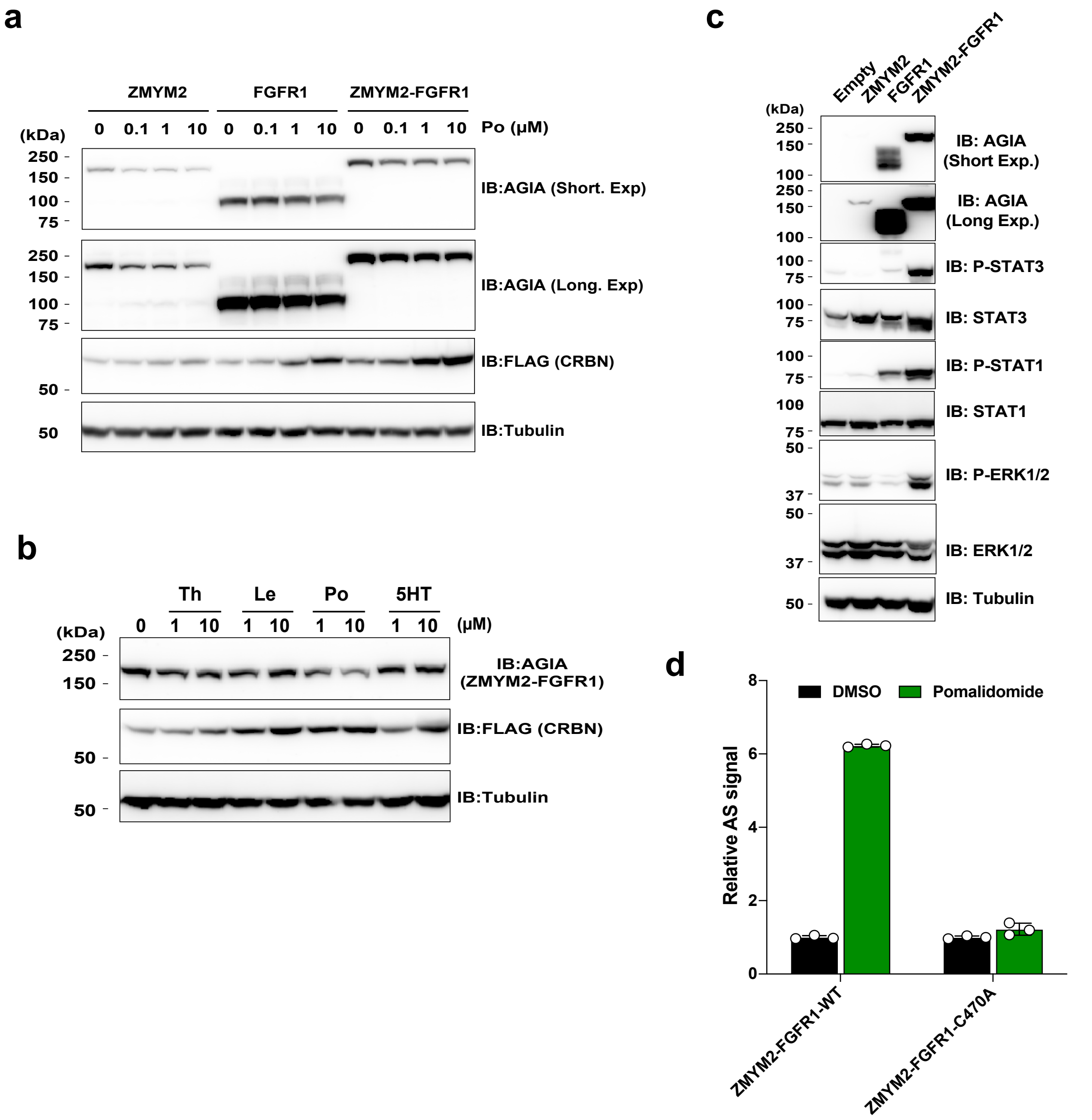

Supplementary Fig. 10. ZMYM2-FGFR1 is a pomalidomide (Po) neo-substrate and activates the FGFR1 signalling pathway. a, Immunoblot analysis of ZMYM2-, FGFR1- or ZMYM2-FGFR1-AGIA protein levels in HEK293T cells expressing ZMYM2-, FGFR1- or ZMYM2-FGFR1-AGIA and FLAG-CRBN treated with DMSO or pomalidomide (Po) for 16 h. The experiment was repeated three times independently with similar results. **b,** Thalidomide and its derivatives (IMiDs) specificity of protein degradation of ZMYM2-FGFR1-AGIA. The protein levels of ZMYM2-FGFR1 in HEK293T cells expressing ZMYM2-FGFR1-AGIA and FLAG-CRBN treated with DMSO, thalidomide (Th), lenalidomide (Le), pomalidomide (Po) or 5-hydroxythalidomide (5HT) for 16 h were analysed by immunoblotting. The experiment was repeated twice independently with similar results. **c,** Immunoblot analysis of phosphorylated proteins by ZMYM2-FGFR1 in HEK293T cells. Cell lysates of HEK293T cells stably expressing empty, AGIA-ZMYM2, -FGFR1 or ZMYM2-FGFR1 were analysed by immunoblot analysis. The experiment was repeated twice independently with similar results. **d,** *In vitro* interaction assay between CRBN and ZMYM2-FGFR1-WT or -C470A. The interaction between bls-CRBN and FLAG-GST-ZMYM2-FGFR1-WT or -C470A in the presence of DMSO or 20 μM pomalidomide (Po) was analysed using the AlphaScreen (AS)-based biochemical assay. Relative AS signals are expressed as luminescence signals relative to that of DMSO, and error bars denote the mean ± standard deviation (independent experiments; n = 3). Source data are provided as a Source data file.

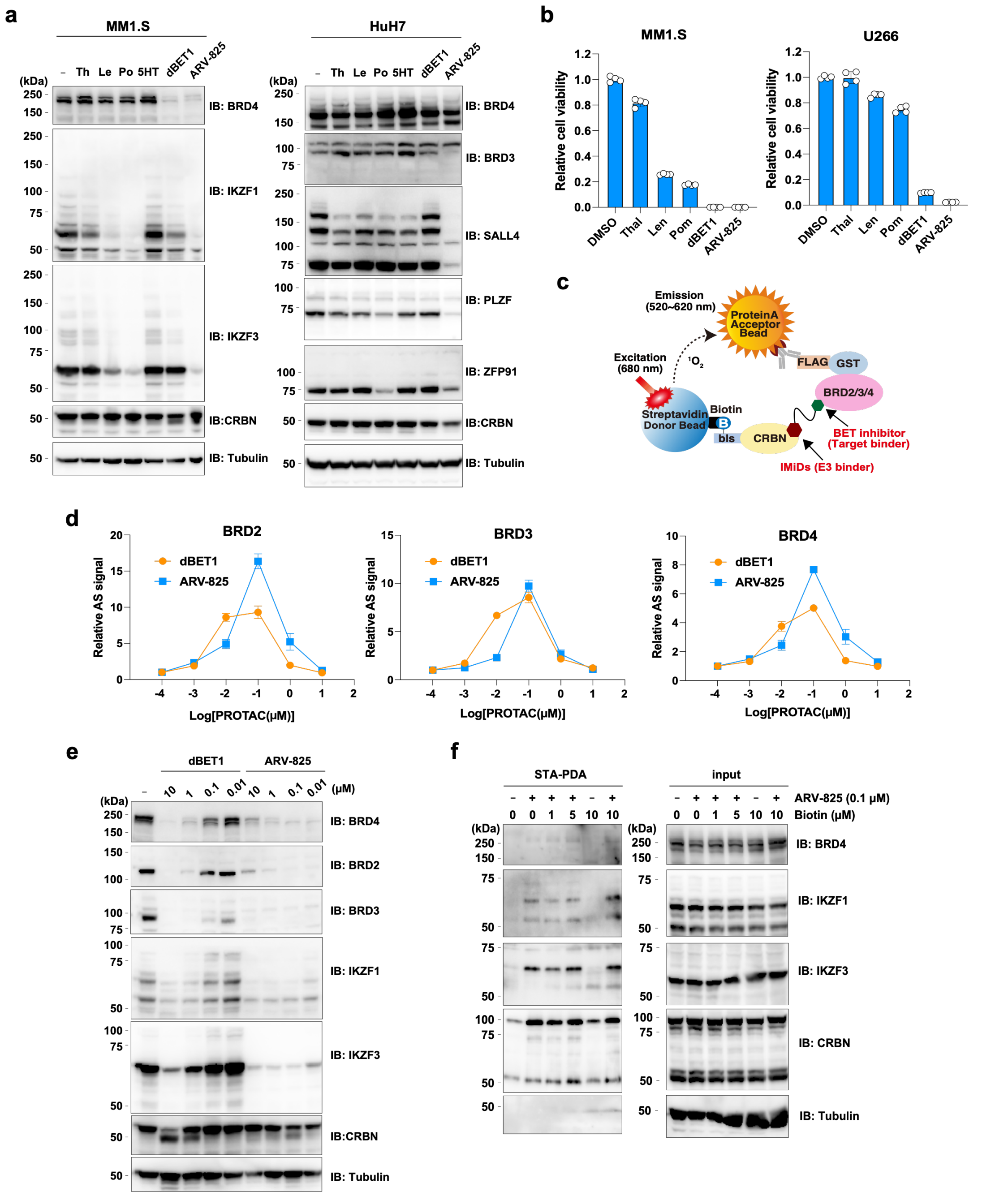

Supplementary Fig. 11. Characterisation of proteolysis-targeting chimaeras (PROTACs) for BRD proteins *in vitro* and in cells. a, Immunoblot analysis of endogenous thalidomide and its derivatives (IMiDs) neo-substrates and BRD family protein levels in MM1.S cells or HuH7 cells treated with DMSO, 20 μM thalidomide (Th), 10 μM lenalidomide (Le), 1 μM pomalidomide (Po), 20 μM 5-hydroxythalidomide (5HT), 1 μM dBET1 or 1 μM ARV-825 for 24 h. The experiment was repeated twice independently with similar results. **b,** Cell viability assay of MM1.S cells and U266 cells treated with IMiDs or PROTACs. MM1.S cells and U266 cells were treated with DMSO, 10 μM thalidomide (Thal), 1 μM lenalidomide (Len), 0.1 μM pomalidomide (Pom), 10 μM dBET1 or 0.1 μM ARV-825 for 6 d and cell viability was detected by Cell-Titer-Glo assay. All relative cell viabilities are expressed as luminescence signals relative to that of DMSO. Error bars denote the standard deviation (biological replicates; n = 4). **c,** Schematic diagram of the AlphaScreen (AS)-based biochemical assay for detecting the PROTAC-dependent interaction between CRBN and BRD proteins. **d,** PROTAC dose-dependent *in vitro* interaction

assay between CRBN and BRD proteins. The interaction between bls-CRBN and FLAG-GST-BRD2, -BRD3, or -BRD4 in the presence of DMSO, dBET1 or ARV-825 (0.0001, 0.001, 0.01, 0.1, 1 or 10 μM) was analysed using the AlphaScreen-based biochemical assay. All relative AlphaScreen (AS) signals are expressed as luminescence signals relative to that of DMSO. Error bars denote the standard deviation (independent experiments; n = 3). **e,** Immunoblot analysis of endogenous IMiD neo-substrates and BRD family protein levels in MM1.S cells treated with DMSO, dBET1 or ARV-825 for 24 h. The experiment was repeated twice independently with similar results. **f,** Biotin dose-dependent analysis of PROTAC-induced biotinylation of neo-substrates and BRD4 in MM1.S cells. MM1.S cells stably expressing AGIA-AirID-CRBN-WT were treated with DMSO or 0.1 μM ARV-825, 0, 1, 5 or 10 μM biotin and 5 μM MG132 for 6 h. Then, the biotinylated proteins were pulled down using streptavidin beads and analysed by immunoblotting. The experiment was repeated twice independently with similar results. Source data are provided as a Source data file.
