# Supplementary material for: A proximity biotinylation-based approach to identify protein-E3 ligase interactions induced by PROTACs and molecular glues
Source: Nat Commun. 2022 Jan 10;13:183. doi: 10.1038/s41467-021-27818-z (PMC8748630; doi:10.1038/s41467-021-27818-z)
Supplement: Supplementary file 3 — Description of Additional Supplementary Files [file 41467_2021_27818_MOESM3_ESM.docx]

File Name: Supplementary Data 1
Description: LC-MS/MS analysis of biotinylated peptides in AirID-CRBN-expressing THP-1 cells treated with DMSO, thalidomide or pomalidomide.

File Name: Supplementary Data 2
Description: LC-MS/MS analysis of biotinylated peptides enriched by using tamavidin 2-REV.

File Name: Supplementary Data 3
Description: LC-MS/MS analysis of biotinylated proteins enriched by using streptavidin.

File Name: Supplementary Data 4
Description: LC-MS/MS analysis of biotinylated peptides enriched by using anti-biotin antibody.

File Name: Supplementary Data 5
Description: LC-MS/MS analysis of biotinylated peptides in AirID-CRBN-expressing MM1.S cells treated with DMSO, thalidomide, lenalidomide, pomalidomide or 5-hydroxythalidomide.

File Name: Supplementary Data 6
Description: LC-MS/MS analysis of biotinylated peptides in AirID-CRBN-expressing HEK293T cells treated with DMSO, thalidomide, lenalidomide, pomalidomide or 5-hydroxythalidomide.

File Name: Supplementary Data 7
Description: LC-MS/MS analysis of biotinylated peptides in AirID-CRBN-expressing HuH7 cells treated with DMSO, thalidomide, lenalidomide, pomalidomide or 5-hydroxythalidomide.

File Name: Supplementary Data 8
Description: LC-MS/MS analysis of biotinylated peptides in AirID-CRBN-expressing IMR32 cells treated with DMSO, thalidomide, lenalidomide, pomalidomide or 5-hydroxythalidomide.

File Name: Supplementary Data 9
Description: LC-MS/MS analysis of biotinylated peptides in AirID-CRBN-expressing IMR32 cells treated with DMSO or pomalidomide.

File Name: Supplementary Data 10
Description: LC-MS/MS analysis of biotinylated peptides in TurboID-CRBN-expressing IMR32 cells treated with DMSO or pomalidomide.

File Name: Supplementary Data 11
Description: LC-MS/MS analysis of biotinylated peptides in AirID-DCAF15-expressing HCT116 cells treated with DMSO or indisulam.

File Name: Supplementary Data 12
Description: LC-MS/MS analysis of biotinylated peptides in AirID-CRBN-expressing MM1.S cells treated with DMSO, pomalidomide or ARV-825.
