# Supplementary material for: A proximity biotinylation-based approach to identify protein-E3 ligase interactions induced by PROTACs and molecular glues
Source: Nat Commun. 2022 Jan 10;13:183. doi: 10.1038/s41467-021-27818-z (PMC8748630; doi:10.1038/s41467-021-27818-z)
Supplement: Supplementary file 16 — Reporting Summary [file 41467_2021_27818_MOESM16_ESM.pdf]

nature research

Corresponding author(s): Tatsuya Sawasaki

Last updated by author(s): Nov 12, 2021

Reporting Summary

Nature Research wishes to improve the reproducibility of the work that we publish. This form provides structure for consistency and transparency in reporting. For further information on Nature Research policies, see our Editorial Policies and the Editorial Policy Checklist.

Statistics

For all statistical analyses, confirm that the following items are present in the figure legend, table legend, main text, or Methods section.

n/a	Confirmed	
☐	☒	The exact sample size (n) for each experimental group/condition, given as a discrete number and unit of measurement
☐	☒	A statement on whether measurements were taken from distinct samples or whether the same sample was measured repeatedly
☐	☒	The statistical test(s) used AND whether they are one- or two-sided<br>*Only common tests should be described solely by name; describe more complex techniques in the Methods section.*
☒	☐	A description of all covariates tested
☐	☒	A description of any assumptions or corrections, such as tests of normality and adjustment for multiple comparisons
☐	☒	A full description of the statistical parameters including central tendency (e.g. means) or other basic estimates (e.g. regression coefficient) AND variation (e.g. standard deviation) or associated estimates of uncertainty (e.g. confidence intervals)
☐	☒	For null hypothesis testing, the test statistic (e.g. F, t, r) with confidence intervals, effect sizes, degrees of freedom and P value noted<br>*Give P values as exact values whenever suitable.*
☒	☐	For Bayesian analysis, information on the choice of priors and Markov chain Monte Carlo settings
☒	☐	For hierarchical and complex designs, identification of the appropriate level for tests and full reporting of outcomes
☒	☐	Estimates of effect sizes (e.g. Cohen's d, Pearson's r), indicating how they were calculated

Our web collection on statistics for biologists contains articles on many of the points above.

Software and code

Policy information about availability of computer code

Data collection	All data collection in this study were performed using softwares attached to each detector.<br>Image Quant LAS 4000 software (GE Healthcare, version 1.1) for chemical luminescent immunoblot.<br>Odyssey Fc Image Studio software (LI-COR Biosciences, version 5.2) for fluorescent immunoblot.<br>Wallac Envision Manager software (ParkinElmer, version 1.12) for AlphaScreen.<br>LightCycler 96 software (Roche, version 1.1) for quantitative-PCR.<br>Glomax 96 software (Promega, version 1.9.3) for CellTiter-Glo.<br>Proteome Discoverer software (Thermo Fisher Scientific, version 2.4.1.15) for LC-MS/MS.
Data analysis	Image analysis was performed using ImageJ software (version 2.1.0).<br>Empiria Studio software (version 1.3) was used for fluorescent immunoblot analysis.<br>Data analysis and significant changes were performed using Excel (version 16.54) or GraphPad Prism 8 (Version 8.4.3).

For manuscripts utilizing custom algorithms or software that are central to the research but not yet described in published literature, software must be made available to editors and reviewers. We strongly encourage code deposition in a community repository (e.g. GitHub). See the Nature Research guidelines for submitting code & software for further information.

Data

Policy information about availability of data

All manuscripts must include a data availability statement. This statement should provide the following information, where applicable:

- Accession codes, unique identifiers, or web links for publicly available datasets
- A list of figures that have associated raw data
- A description of any restrictions on data availability

The MS proteomics data have been provided in Supplementary Data 1–12 and deposited to the ProteomeXchange Consortium via the jPOST partner res pository with the dataset identifiers PXD028754 [http://proteomecentral.proteomexchange.org/cgi/GetDataset?ID=PXD028754] (three enrichment methods using AirID-CRBN-expressing MM1.S cells), PXD028755 [http://proteomecentral.proteomexchange.org/cgi/GetDataset?ID=PXD028755] (neo-substrate selectivity of IMiDs using AirID-CRBN-expressing MM1.S cells), PXD028756 [http://proteomecentral.proteomexchange.org/cgi/GetDataset?ID=PXD028756] (LC-MS/MS analysis of IMiDs-dependent biotinylated peptides using AirID-CRBN-expressing HEK293T cells), PXD028757 [http://proteomecentral.proteomexchange.org/cgi/GetDataset?ID=PXD028757] (LC-MS/MS analysis of IMiDs-dependent biotinylated peptides using AirID-CRBN-expressing HuH7 cells), PXD028758 [http://proteomecentral.proteomexchange.org/cgi/GetDataset?ID=PXD028758] (LC-MS/MS analysis of IMiDs-dependent biotinylated peptides using AirID-CRBN-expressing IMR32 cells), PXD028760 [http://proteomecentral.proteomexchange.org/cgi/GetDataset?ID=PXD028760] (LC-MS/MS analysis of IMiDs-dependent biotinylated peptides using AirID-CRBN-expressing THP-1 cells), PXD028761[http://proteomecentral.proteomexchange.org/cgi/GetDataset?ID=PXD028761] (Validation of AirID and TurboID using AirID-CRBN- or TurboID-CRBN-expressing IMR32 cells), PXD028762[http://proteomecentral.proteomexchange.org/cgi/GetDataset?ID=PXD028762] (LC-MS/MS analysis of Indisulam-dependent biotinylated peptides using AirID-DCAF15-expressing HCT116 cells) and PXD028763 [http://proteomecentral.proteomexchange.org/cgi/GetDataset?ID=PXD028763] (LC-MS/MS analysis of PROTACs-dependent biotinylated peptides using AirID-CRBN-expressing MM1.S cells). All data supporting the findings of this study are provided in the main text and supplementary information.

Field-specific reporting

Please select the one below that is the best fit for your research. If you are not sure, read the appropriate sections before making your selection.

[x] Life sciences [ ] Behavioural & social sciences [ ] Ecological, evolutionary & environmental sciences

For a reference copy of the document with all sections, see nature.com/documents/nr-reporting-summary-flat.pdf

Life sciences study design

All studies must disclose on these points even when the disclosure is negative.

Sample size	For proteasomal degradation assays and streptavidin pull-down assays, we have chosen more than two independent experiments as sample sizes based on our previous papers in same research field (Yamanaka et al., EMBO J. 2021;40(4):e105375, Furihata et al., Nat. Commun. 2020;11(1):457 and Kido et al., eLife 2020;9:e54983) and traditional experimental approach in biochemical and cellular experiments. For quantitative experiments, such as LC-MS/MS and qPCR, samples were prepared in at least triplicates.
Data exclusions	No data were excluded from the analyses.
Replication	Proteasomal degradation assays were performed three times independently with similar results. Streptavidin pull-down assays were performed at least twice independently with similar results. In the other experiments including mass spectrometry experiments, replication was performed as indicated in the methods section or figure legend.
Randomization	Randomization was not relevant because there is no allocation of samples/organisms/participants involved in this study.
Blinding	Blinding was not necessary because there is no group allocation involved in this study.

Reporting for specific materials, systems and methods

We require information from authors about some types of materials, experimental systems and methods used in many studies. Here, indicate whether each material, system or method listed is relevant to your study. If you are not sure if a list item applies to your research, read the appropriate section before selecting a response.

Materials & experimental systems

n/a	Involved in the study	
[ ]	[x]	Antibodies
[ ]	[x]	Eukaryotic cell lines
[x]	[ ]	Palaeontology and archaeology
[x]	[ ]	Animals and other organisms
[x]	[ ]	Human research participants
[x]	[ ]	Clinical data
[x]	[ ]	Dual use research of concern

Methods

n/a	Involved in the study	
[x]	[ ]	ChIP-seq
[x]	[ ]	Flow cytometry
[x]	[ ]	MRI-based neuroimaging

Antibodies

Antibodies used	Anti-FLAG mouse mAb clone M2 (HRP-conjugated, Sigma-Aldrich, # A8592, 1:5000), anti-AGIA rabbit mAb (HRP-conjugated, produced in our laboratory, 1:5000), and anti-Myc-tag mAb clone 9B11 (HRP-conjugated, Cell Signaling Technology, #2040, 1:1000) were used to detect epitope-tagged proteins. Anti-α-tubulin rabbit pAb (HRP-conjugated, MBL, # PM054-7, 1:5000) and anti-α-tubulin mouse mAb (LI-COR Biosciences, # 926-42213, 1:1000) was used to detect α-tubulin. Biotinylated proteins were detected by anti-biotin goat pAb (HRP-conjugated, Cell Signaling Technology, # 7075, 1:3000). Anti-CRBN rabbit mAb clone D8H3S (Cell Signaling Technology, # 71810, 1:1000), anti-IKZF1/Ikaros rabbit mAb clone D6N9Y (Cell Signaling Technology, # 14859, 1:1000), anti-IKZF3/Aiolos rabbit mAb clone D1C1E (Cell Signaling Technology, #15103, 1:1000), anti-GSPT1 rabbit pAb (Cell Signaling Technology, # 14980, 1:1000), anti-phospho-STAT1 rabbit mAb clone 58D6 (Cell Signaling Technology, #9167, 1:1000), anti-STAT1 mouse mAb clone 9H2 (Cell Signaling Technology, # 9176, 1:1000), anti-phospho-STAT3 rabbit mAb clone D3A7 (Cell Signaling Technology, #9145, 1:1000), anti-STAT3 rabbit pAb (Cell Signaling Technology, # 9132, 1:1000), anti-phospho-ERK1/2 rabbit mAb clone 197G2 (Cell Signaling Technology, #4377, 1:1000), anti-ERK1/2 rabbit mAb clone 137F5 (Cell Signaling Technology, # 4695, 1:1000), anti-SALL4 rabbit pAb (Abcam, # ab29112, 1: 1000), anti-SALL4 mouse mAb clone EE-30 (Santa Cruz Biotechnology, # sc-101147, 1:500), anti-PLZF goat pAb (R&D System, AF2944, 1:1000), anti-ZNF687 rabbit pAb (Bethyl Laboratories, # A303-278A, 1:1000), anti-WIZ rabbit pAb (Bethyl Laboratories, # A305-864A, 1:1000), anti-ZFP91 rabbit pAb (Bethyl Laboratories, # A303-245A, 1:1000), anti-BRD4 rabbit pAb (Bethyl Laboratories, # A301-985A, 1:1000), anti-BRD2 rabbit pAb (Bethyl Laboratories, # A302-583A, 1:1000), anti-ZMYM2 rabbit pAb (Gene Tex, # GTX105550, 1:1000), anti-ZMYM2 rabbit pAb (Gene Tex, # GTX31821, 1:1000), anti-CK1α rabbit mAb clone EPR1961(2) (Abcam, ab108296), anti-BRD3 rabbit pAb (Proteintech, # 11859-1-AP, 1:1000) and anti-RBM39 rabbit pAb (Sigma-Aldrich, # HPA001591, 1:1000) were used as primary antibodies. Anti-rabbit IgG (HRP-conjugated, Cell Signaling Technology, # 7074, 1:10000), anti-mouse IgG (HRP-conjugated, Cell Signaling Technology, # 7076, 1:10000), anti-goat IgG (HRP-conjugated, Invitrogen/Thermo Fisher Scientific, #81-1620, 1:10000), IRDye 800CW goat anti-rabbit IgG (LI-COR Biosciences, # 925-32211, 1:10000) and IRDye 680RD goat anti-mouse IgG (LI-COR Biosciences, # 925-68070, 1:10000) were used as secondary antibodies.
Validation	All primary antibodies in this study were purchased from commercial companies. All of these antibodies have been validated for the human species as described on the supplier's websites.<br>The anti-CRBN rabbit mAb (Cell Signaling Technology, # 71810, 1:1000), anti-IKZF1/Ikaros rabbit mAb (Cell Signaling Technology, # 14859, 1:1000), anti-IKZF3/Aiolos rabbit mAb (Cell Signaling Technology, #15103, 1:1000), anti-GSPT1 rabbit pAb (Cell Signaling Technology, # 14980, 1:1000), anti-phospho-STAT1 rabbit mAb (Cell Signaling Technology, #9167, 1:1000), anti-STAT1 mouse mAb (Cell Signaling Technology, # 9176, 1:1000), anti-phospho-STAT3 rabbit mAb (Cell Signaling Technology, #9145, 1:1000), anti-STAT3 rabbit Ab (Cell Signaling Technology, # 9132, 1:1000), anti-phospho-ERK1/2 rabbit mAb (Cell Signaling Technology, #4377, 1:1000), anti-ERK1/2 rabbit mAb (Cell Signaling Technology, # 4695, 1:1000) have been validated for detection of human species by immunoblot analysis as described on Cell Signaling Technology websites for specific antibodies.<br>The anti-SALL4 rabbit pAb (Abcam, # ab29112, 1: 1000), anti-SALL4 mouse mAb (Santa Cruz Biotechnology, # sc-101147, 1:500), anti-PLZF goat pAb (R&D System, AF2944, 1:1000), anti-ZNF687 rabbit pAb (Bethyl Laboratories, # A303-278A, 1:1000), anti-WIZ rabbit pAb (Bethyl Laboratories, # A305-864A, 1:1000), anti-ZFP91 rabbit pAb (Bethyl Laboratories, # A303-245A, 1:1000), anti-BRD4 rabbit pAb (Bethyl Laboratories, # A301-985A, 1:1000), anti-BRD2 rabbit pAb (Bethyl Laboratories, # A302-583A, 1:1000), anti-ZMYM2 rabbit pAb (Gene Tex, # GTX105550, 1:1000), anti-ZMYM2 rabbit pAb (Gene Tex, # GTX31821, 1:1000), anti-CK1α rabbit mAb (Abcam, ab108296), anti-BRD3 rabbit pAb (Proteintech, # 11859-1-AP, 1:1000) and anti-RBM39 rabbit pAb (Sigma-Aldrich, # HPA001591, 1:1000) have been validated for detection of human species by immunoblot analysis as described on each supplier's website for specific antibodies.<br>The anti-FLAG mouse mAb (HRP-conjugated, Sigma-Aldrich, # A8592, 1:5000) and anti-Myc-tag mAb (HRP-conjugated, Cell Signaling Technology, #2040, 1:1000) have been validated for detection of epitope-tagged proteins by immunoblot analysis as described on each supplier's website for specific antibodies.<br>The anti-α-tubulin rabbit pAb (HRP-conjugated, MBL, # PM054-7, 1:5000) and anti-α-tubulin mouse mAb (LI-COR Biosciences, # 926-42213, 1:1000) have been validated for detection of human α-tubulin by immunoblot analysis as described on each supplier's website.<br>The anti-biotin goat pAb (HRP-conjugated, Cell Signaling Technology, # 7075, 1:3000) has been validated for detection of biotinylated proteins by immunoblot analysis on Cell Signaling Technology website.<br>The anti-rabbit IgG (HRP-conjugated, Cell Signaling Technology, # 7074, 1:10000), anti-mouse IgG (HRP-conjugated, Cell Signaling Technology, # 7076, 1:10000), anti-goat IgG (HRP-conjugated, Invitrogen/Thermo Fisher Scientific, #81-1620, 1:10000), IRDye 800CW goat anti-rabbit IgG (LI-COR Biosciences, # 925-32211, 1:10000) and IRDye 680RD goat anti-mouse IgG (LI-COR Biosciences, # 925-68070, 1:10000) have been validated as secondary antibody for immunoblot analysis as described on each supplier's website.<br>The AGIA antibody has been validated in published paper (Yano, et al., PLoS ONE 11: e0156716) cited in Methods.

Eukaryotic cell lines

Policy information about cell lines

Cell line source(s)	MM1.S, U266 and HEK293T cell lines were purchased from American Type Culture Collection (ATCC). HEK293T-CRBN-KO cell line was generated from HEK293T cell line purchased from Riken BioResource Research Center (Riken BRC) (Yamanaka et al., Commun. Biol. 2020;3:515). HCT116 and THP-1 cell lines were purchased from Riken BRC. IMR32 cell line was purchased from Japanese Collection of Research Bioresources Cell Bank (JCRB Cell Bank). HuH7 cell line originally obtained from JCRB Cell Bank, was kindly provided from Prof. T. Okamoto (Osaka University).
Authentication	All cell lines were used for each experiment between passage number 5 and 15 to avoid changes in the cell line's properties. In addition, all cell lines were authenticated by morphological appearances by careful observation.
Mycoplasma contamination	All cell lines used in this study were tested negative for mycoplasma.
Commonly misidentified lines (See ICLAC register)	No commonly misidentified cell lines were used in this study.
